# How to use and report data on D‐dimer testing in the COVID‐19 era?

**DOI:** 10.1002/rth2.12817

**Published:** 2022-11-23

**Authors:** Litao Zhang, Xiaohui Liu, Zhenlu Zhang

**Affiliations:** ^1^ Clinical Laboratory Wuhan Asia General Hospital Affiliated to Wuhan University of Science and Technology Wuhan China; ^2^ Laboratory Medicine Wuhan Asia Heart Hospital Wuhan China

**Keywords:** COVID‐19, D‐dimer, D‐dimer ratio, report, standardization

We read with interest the study published in the recent issue by Woller and colleagues,^1^ entitled “Biomarker derived risk scores predict venous thromboembolism and major bleeding among patients with COVID‐19.” We appreciated that they introduced D‐dimer to the study to improve the predictiveness of the biomarkers score. However, we noticed that there was a little deficiency in the use and report of D‐dimer testing in this article.

It is well known that the standardization and harmonization of D‐dimer testing are poor, and fibrinogen equivalent units (FEU) and D‐dimer units (DDU) are the two units commonly used to report D‐dimer levels. However, there is a huge difference in D‐dimer results between these two reporting systems.^2^ Therefore, the D‐dimer results among institutions are significantly different and have little comparability. In Woller's study, the participants were assigned into three categories based on D‐dimer levels: D‐dimer <0.5, 0.5–2.0, and greater than 2.0 μg/ml. However, using the absolute D‐dimer values in the analysis would limit the use of the biomarker score model for the hospitals using different D‐dimer reagents. Thus, we suggest using the D‐dimer ratio (DDR) instead of absolute D‐dimer values to stratify the patients in their study. DDR means the ratio of the D‐dimer value to the upper limit of the normal range (ULN) for the current D‐dimer assay (DDR = D‐dimer/ULN). Using the DDR reporting system, the three categories in Woller's study could be translated into DDR less than 1.0, 1.0–4.0, and greater than 4.0, with the ULN of 0.5 μg/ml stated in the study. DDR can show the proportional level of D‐dimer elevation, independent of the unit type used, and accounts for the cutoff value used. This is a simple and helpful transformation to apply the predictive model more widely. The DDR had been employed in several multicenter clinical trials on COVID‐19 (the RAPID, ATTACC, ACTIV‐4a, and REMAP‐CAP studies) to ensure comparable D‐dimer results across institutions.^3^, ^4^

Additionally, the details of the D‐dimer assay in Woller's study were not described clearly. We speculated that it would be an FEU reagent since the ULN is 0.5 μg/ml, which should be declared in the paper. Furthermore, the authors should also provide the analytical performance of the D‐dimer assay because it is important for readers to know the potential statistical bias of the predictive model. We suggest that the authors refer to the recommendations from a previous official communication (Thachil J et al.) from the ISTH Scientific and Standardization Subcommittee on fibrinolysis.^5^

## RELATIONSHIP DISCLOSURE

The authors declare that they have no conflicts of interest regarding this article.

## AUTHOR CONTRIBUTIONS

LZ and XL drafted the manuscript, and ZZ participated in discussion and critical editing of the manuscript.

## FUNDING INFORMATION

This work was supported by the Hubei Provincial Nature Science Foundation of China (2020CFB865) and 2020 Wuhan Young & Middle‐age Medical Backbone Training Programme.
